# Association between oral health status and functional independence measure on admission in convalescent hospitalized patients

**DOI:** 10.1186/s12903-023-03667-8

**Published:** 2024-01-09

**Authors:** Ryuzo Hara, Naoki Todayama, Tomohiro Tabata, Tomoko Mukai, Yukiko Hatanaka, Masataka Watanabe, Miki Kuwazawa, Shouji Hironaka, Nobuyuki Kawate, Junichi Furuya

**Affiliations:** 1 Department of Oral Function Management, Graduate School of Dentistry, Showa University, Ota, Tokyo Japan; 2 Division of Oral Function Management, Department of Oral Health Management, School of Dentistry, Showa University, Ota, Tokyo Japan; 3 Fujigaoka Hospital Hospitaly Dentistry, Yokohama, Kanagawa Japan; 4 Department of Hygiene and Oral Health, School of Dentistry, Showa University, Shinagawa, Tokyo Japan; 5 Department of Rehabilitation Medicine, School of Medicine, Showa University, Shinagawa, Tokyo Japan

**Keywords:** Oral health management, Oral health assessment tool, Functional independence measure, Rehabilitation, Hospital, Multidisciplinary team

## Abstract

**Background:**

Oral health management has become increasingly important for acute inpatients. Older patients often require extended periods of medical care, and oral health management is necessary in the convalescent period following the acute period. During the convalescent period, oral health management remains unclear as convalescent hospitals have limited dental resources, and effective dental care must be provided if the objective of hospitalization is to improve life functions. This study aimed to clarify the relationship between daily functioning and oral health status at the time of admission to a convalescent hospital to aid in improving daily functioning in the convalescent period.

**Methods:**

We included patients admitted to the rehabilitation department of a specific convalescent hospital from January to December 2021. A total of 375 patients were included in the study, with complete data records. At admission, we gathered information from the medical records, including the patient’s age, sex, primary disease, Charlson Comorbidity Index, Mini Nutritional Assessment Short-Form (MNA-SF), Functional Oral Intake Scale (FOIS), Functional Independence Measure (FIM), number of teeth, and Oral Health Assessment Tool (OHAT). Statistical analysis was conducted using SPSS Ver. 27, with a significance level of 5%.

**Results:**

The mean age of the 375 participants (189 men and 186 women) was 75.0 ± 12.1 years (range, 42–97 years), and over 80% were > 65 years. About 30% of major diseases could be attributed to strokes and fractures, followed by spinal cord and spine diseases. In non-stroke patients, multiple regression analysis using FIM motor, FIM cognitive, and FIM and OHAT total scores as objective variables showed that higher total scores of MNA-SF, FOIS, and lower modified Rankin Scale and OHAT were significantly associated with better FIMs. Lower OHAT scores were significantly associated with lower FOIS and MNA-SF scores, male sex, having fewer teeth, and poor dietary patterns.

**Conclusions:**

The convalescent period is an opportune time to provide intensive dental care due to the generally stable condition and extended hospital stay. Our results suggest that oral health management, such as dysphagia rehabilitation and denture treatment, is important for maintaining and improving independence, a key objective of convalescent rehabilitation, and malnutrition improvement.

## Background

Older adults are susceptible to cerebrovascular diseases, disuse syndrome, and bone fractures [1], and these diseases often require extended periods of medical treatment. After receiving treatment for these diseases, patients are often transferred to convalescent hospitals for rehabilitation. They then continue treatment during the living phase at home or in nursing homes. Seamless oral health management that links these acute, convalescent, and living phases is considered necessary [2].

Convalescent hospitals play an essential role in connecting the acute phase with the living phase. Convalescent hospitals restore independence through intensive rehabilitation so patients can return to their homes or nursing homes and live independently [3]. It is said that the appropriate time for discharge from a convalescent hospital is when the patient becomes independent in activities of daily living (ADL) or when there is no improvement in function despite training [4, 5]. Although individual rehabilitation by physical therapists, occupational therapists, speech therapists, and other professionals is essential to promoting independence, comprehensive management through multidisciplinary cooperation plays a key role in improving the level of independence in hospitals of convalescence [6]. Oral health management by dentists may also contribute to such multidisciplinary collaboration to improve ADL in the convalescent period through oral care and recovery of eating patterns and occlusal support [7].

Patients are transferred to convalescent hospitals after treatment for stroke and other illnesses is completed in the acute phase. Oral health management for stroke patients in acute care hospitals is often inadequate. Inadequate oral hygiene management is directly linked to the deterioration of a patient’s overall health. Research demonstrates that adequate oral hygiene not only improves the oral intake status of patients with acute stroke but also significantly reduces the incidence of pneumonia [8]. Although the effectiveness of oral health management through multidisciplinary collaboration has been demonstrated in the acute phase, even when this is done appropriately, the improvement is limited, partly due to the short hospitalization period in acute care hospitals [9]. Therefore, oral health management should be initiated immediately after hospitalization for convalescence. In addition, hospitalization tends to be relatively prolonged in the recovery phase due to intensive rehabilitation aimed at returning patients home. Furthermore, it is assumed that after returning home, oral health management will mainly be provided through home-visit dental treatments, which are often restricted; therefore, convalescent hospitalization before returning home is an opportune time to provide dental treatment.

A previous study has revealed the importance of oral hygiene management in stroke patients in convalescent hospitals, and early detection of poor oral hygiene in patients admitted to convalescent hospitals may improve the patient’s general condition [10]. In addition, stroke patients are often tube-fed after being transferred from acute care hospitals, and many are anorexic. The poor oral health condition of patients with anorexia suggests the necessity of oral hygiene management in convalescent institutions [11] and highlights the association between tongue coating and the number of teeth [12]. The role of dentistry extends beyond oral hygiene management to include oral function management, which includes tooth and tongue functions that are directly related to eating [13]. It has been shown that stroke patients in convalescent hospitals with poor oral intake status have lower tongue pressure and higher OHAT scores, indicating poor overall oral health status [14, 15]. Furthermore, oral hygiene management and the presence of dental professionals have been found to be associated with regaining independence, which is the primary objective of recovery hospitalization [16–18].

However, the oral health status and the relevance of managing the oral health of patients in convalescent hospitals are still unclear. The limited resources for dentistry during the convalescent period necessitate research leading to the efficient and effective provision of dental care that could improve the quality of life. Moreover, it is important to investigate the oral health status during admission to a convalescent hospital in relation to ADL because regaining independence during the first 2 weeks after admission is related to the level of independence at discharge [19]. Therefore, this study aimed to clarify the relationship between lifestyle function and oral health status at the time of admission to a convalescent hospital in improving lifestyle function from the standpoint of dentistry.

## Methods

### Participants

Of 398 consecutive patients who were admitted to the rehabilitation department of a particular convalescent hospital between January and December 2021, 375 participants were included in the study (mean age 75.2 ± 11.9 years, 189 men, 186 women). The patients’ ages ranged from 42 to 97 years, with no exclusion criteria based on age. Twenty-three participants were excluded based on the following exclusion criteria: early discharge within 1 week and inability to obtain data, missing data, and refusal of dental examination upon admission. The hospital under study was the only university-affiliated convalescent hospital, a private hospital with 197 beds. Any patient with a disease requiring rehabilitation could be admitted. Data at the time of admission were obtained only once, within 10 days of admission. Upon admission, we extracted comprehensive systemic and oral health information from the medical records and conducted a cross-sectional investigation. Before commencing the study, we obtained informed consent from all participants using an opt-out method, ensuring their understanding and agreement to participate. Data for this study were collected by several calibrated dentists. This study was conducted with the approval of the Clinical Trial Review Committee of Showa University Fujigaoka Hospital (Approval Number: F2020C158).

### Data collection and variables

From the medical records, we extracted information regarding age at admission, sex, primary disease, comorbidities, Functional Independence Measure (FIM), modified Rankin Scale (mRS), Functional Oral Intake Scale (FOIS), clinical severity classification of dysphagia (DSS), Mini Nutritional Assessment Short-Form (MNA-SF), number of present teeth, functional teeth, and Oral Health Assessment Tool (OHAT).

The primary diagnosis was extracted from the medical records at admission to the convalescent hospital. Stroke was classified as follows: 0: no stroke or 1: stroke. The presence of fracture was classified as 0: no fracture, 1: fracture. Comorbidities were scored using the Charlson Comorbidity Index (CCI) [20, 21]. The level of independence at admission was assessed by rehabilitation physicians using the FIM [22]. The FIM is an ADL assessment method developed by Granger et al. in 1983 [23]. The assessment items are motor and cognitive items (18 items), and each item is rated on a 7-point scale from 1 to 7. The higher the score, the higher the level of independence. The severity of the stroke was evaluated using the mRS [24]. mRS is used to evaluate stroke severity and is rated on a 7-point scale from 0 to 6, with higher values indicating a more severe stroke [25]. FOIS, a nutritional intake method, evaluates oral intake on a 7-point scale from level 1 to level 7 [26]. The higher the score, the better the swallowing function. Swallowing function was evaluated using the DSS, a clinical severity classification for dysphagia developed by Saito et al. in 1999 [27]. The DSS is a seven-level severity scale (1 to 7); the higher the number, the more normal the patient’s condition. Nutritional status was assessed using the MNA-SF, a nutritional screening tool specifically designed for older adults [28–30]. The MNA-SF consists of six items, each scored in the range of 0–2 or 3 points, with higher total scores indicating better nutritional status. The number of teeth present was defined as those excluding pontics of bridges and missing prosthetics such as dentures and implants. The number of functional teeth was defined as all teeth, including pontics, dentures, and implants of bridges, excluding teeth with degree III or higher mobility and C4 teeth. The OHAT [31], which indicates comprehensive oral health status, is a three-point scale (0–2) that evaluates eight items: lips, tongue, gums and tissues, saliva, natural teeth, dentures, oral cleanliness, and dental pain. The higher the total score, the worse the oral health status.

### Statistical analysis

First, the correlation between oral health status and general health status was examined using Spearman’s correlation analysis. Then, multiple regression analysis was conducted using FIM motor, FIM cognitive, and FIM total score as objective variables and factors related to the oral cavity and the whole body as explanatory variables. Multiple regression analysis was also performed using the OHAT total score as the objective variable and factors related to oral status and the whole body as explanatory variables. Statistical analysis was conducted using SPSS Ver. 27 (IBM Japan), with a significance level of 5% for all statistical procedures.

## Results

### Participants characteristics

**Fig. 1:**
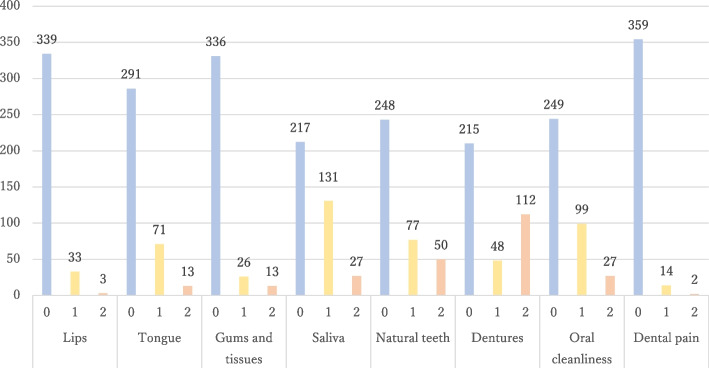
Distribution of OHAT. Figure Legend. Healthy, 1- Oral Changes, and 2- Unhealthy

Table 1 shows the overall characteristics of the study participants. The mean age of the participants was 75.2 years. A total of 322 participants were older adults, aged 65 years or older, and the majority had some kind of systemic disease, such as stroke, fracture, or spinal cord disease, and were admitted to a convalescent hospital for the purpose of post-acute rehabilitation. Approximately 30% of patients suffered from stroke or fracture, respectively, followed by patients with spinal cord disease or disuse syndrome. CCI was low at 1.5 points, and the general condition was low-risk. The FIM was relatively high on the cognitive items at 28.3 out of 35, but low on the motor items at 49.6 out of 91, resulting in a slightly lower total score of 77.9 out of 126. The median mRS was 4.0, and mRS scores most often demonstrated moderate to severe disability with difficulty walking and fulfilling physical needs without assistance. As per FOIS, many patients ate regular meals. As per DSS, few patients had aspiration, but several had oral problems. Many patients with malnutrition were identified as per MNA-SF. The oral status of the patients was 18.3 teeth (number of present teeth) and 25.0 functional teeth. The OHAT total score was low at 2.6 points. Figure 1 shows the distribution of OHAT subitems. More patients had “oral changes” and “unhealthy” categories marked for saliva, natural teeth, dentures, and oral cleanliness than other items.

**Table 1:** Participants characteristics

	Mean ± SD	Median (Q1-Q3)	N (%)
Age	75.2 ± 11.9	78.0 (70.0–83.0)	–
Main disease			
Stroke	–	–	112 (29.9)
Bone fracture	–	–	123 (32.8)
Spinal cord disease	–	–	52 (13.9)
Disuse syndrome	–	–	44 (11.7)
Limb amputation	–	–	14 (3.7)
Parkinson’s and neuromuscular disease	–	–	5 (1.3)
Others	–	–	25 (6.7)
CCI	1.5 ± 1.5	1.0 (0.0–2.0)	–
FIM			
Motor	49.6 ± 21.4	32.0 (31.0–65.5)	–
Cognitive	28.3 ± 8.3	32.0 (25.0–35.0)	–
Total score	77.9 ± 27.3	83.0 (56.0–99.0)	–
mRS	–	4.0 (4.0–4.0)	–
FOIS	–	7.0 (6.0–7.0)	–
DSS	–	5.0 (5.0–7.0)	–
MNA-SF	9.3 ± 2.6	10.0 (8.0–11.0)	–
Number of teeth	18.3 ± 9.5	21.0 (11.0–26.0)	–
Functional teeth	25.0 ± 6.1	27.0 (24.0–28.0)	–
OHAT	2.6 ± 2.6	2.0 (0.0–4.0)	–

### Univariate analysis of independence and oral health and general health status

Table 2 shows the results of the correlation analysis for each item: the FIM motor, FIM cognitive, and FIM total scores. All showed significant negative correlations with age, presence of stroke, CCI, mRS, and OHAT total score, and significant positive correlations with the presence of fracture, FOIS, DSS, and MNA-SF. Considering these results and their clinical significance, multiple regression analysis was performed using FIM motor items, cognitive items, and total scores as objective variables.

**Table 2:** Correlation coefficients between FIM motor, FIM cognitive, FIM total score, and each parameter (Spearman’s Correlation Analysis)

	FIM motor	FIM cognitive	FIM total score
Age	−0.146**	−0.163**	− 0.173**
Sex	0.071	−0.003	0.056
Stroke	−0.213**	−0.442**	− 0.271**
Bone fracture	0.213**	0.274**	0.232**
CCI	−0.163**	−0.109*	− 0.165**
mRS	−0.718**	− 0.398**	− 0.692**
FOIS	0.532**	0.511**	0.552**
DSS	0.499**	0.438**	0.510**
MNA-SF	0.436**	0.340**	0.448**
Number of present teeth	0.099	0.076	0.106*
Number of Functional teeth	0.095	0.082	0.093
OHAT total score	−0.269**	−0.232**	−0.277**

*p < 0.05, **p < 0.01

### Multivariate analysis by the level of independence and oral/systemic associations

Tables 3, 4 and 5 show the results of multiple regression analysis of FIM motor, FIM cognitive, and FIM total scores. All showed significant negative associations with the presence or absence of stroke, mRS, and OHAT total scores and significant positive associations with FOIS and MNA-SF. Patients with no stroke, less severe stroke, good swallowing function, good nutritional status, and good oral health had higher FIM scores.

**Table 3:** Multiple regression analysis (Objective variable: FIM motor)

Explanatory Variables	B	SE	β	*p*-Value	95% CI	VIF
Age	− 0.037	0.067	− 0.021	0.878	− 0.168 to 0.094	1.340
Sex	−0.567	1.479	−0.013	0.702	−3.475 to 2.342	1.175
Stroke*	−4.250	1.723	−0.091	0.014*	−7.639 to −0.861	1.324
Bone fracture	1.920	1.703	0.042	0.260	−1.429 to 5.269	1.376
mRS*	−17.611	1.225	−0.539	< 0.001*	−20.021 to − 15.201	1.377
CCI	−0.750	0.466	−0.054	0.108	−1.666 to 0.166	1.095
FOIS*	2.352	0.530	0.177	< 0.001*	1.309 to 3.395	1.557
MNA-SF*	1.591	0.287	0.194	< 0.001*	1.027 to 2.155	1.202
Number of present teeth	−0.021	0.081	− 0.009	0.799	− 0.180 to 0.139	1.270
OHAT total score*	−0.677	0.289	−0.082	0.020*	−1.245 to − 0.109	1.192

*p < 0.05, Sex (0=man, 1=women) Stroke (0=not Stroke, 1=Stroke) Bone fractures (0=not Bone fractures, 1=Bone fractures)

**Table 4:** Multiple regression analysis (Objective variable: FIM cognitive)

Explanatory Variables	B	SE	β	p-Value	95% CI	VIF
Age	−0.026	0.031	−0.038	0.392	−0.086 to 0.034	1.340
Sex	−1.114	0.676	−0.068	0.100	−2.445 to 0.216	1.175
Stroke*	−6.132	0.788	−0.339	< 0.001*	−7.683 to −4.582	1.324
Bone fracture	−0.038	0.779	−0.002	0.961	−1.570 to 11.494	1.376
mRS*	−1.524	0.560	−0.121	0.007*	−2.627 to −0.422	1.377
CCI	−0.183	0.213	−0.034	0.390	−0.603 to 0.236	1.095
FOIS*	1.552	0.243	0.302	< 0.001*	1.075 to 2.029	1.557
MNA-SF*	0.647	0.131	0.204	< 0.001*	0.389 to 0.905	1.202
Number of present teeth	0.030	0.037	0.034	0.422	−0.043 to 0.103	1.270
OHAT total score*	−0.398	0.132	−0.125	0.003*	−0.658 to − 0.139	1.192

*p < 0.05, Sex (0=man, 1=women) Stroke (0=not Stroke, 1=Stroke) Bone fractures (0=not Bone fractures, 1=Bone fractures)

**Table 5:** Multiple regression analysis (Objective variable: FIM total score)

Explanatory Variables	B	SE	β	p-Value	95% CI	VIF
Age	− 0.063	0.082	− 0.028	0.442	− 0.225 to 0.099	1.340
Sex	−1.681	1.824	−0.031	0.357	−5.268 to 1.906	1.175
Stroke*	−10.382	2.125	−0.174	< 0.001*	−14.562 to −6.202	1.324
Bone fracture	1.882	2.100	0.032	0.371	−2.248 to 6.012	1.376
mRS*	−19.136	1.511	−0.459	< 0.001*	−22.107 to − 16.164	1.377
CCI	−0.934	0.575	−0.053	0.105	−2.064 to 0.196	1.095
FOIS*	3.904	0.654	0.230	< 0.001*	2.618 to 5.190	1.557
MNA-SF*	2.238	0.354	0.214	< 0.001*	1.542 to 2.934	1.202
Number of present teeth	0.009	0.100	0.003	0.927	−0.188 to 0.206	1.270
OHAT total score*	−1.076	0.356	−0.102	0.003*	−1.776 to −0.375	1.192

*p < 0.05, Sex (0 = man, 1 = women) Stroke (0 = not Stroke, 1 = Stroke) Bone fractures (0 = not Bone fractures, 1 = Bone fractures)

### Multivariate analysis of Oral health care and Oral-systemic relationships

Table 6 shows the results of multiple regression analysis with the total OHAT score as the objective variable. OHAT total score was negatively associated with sex, FOIS, MNA-SF, and number of teeth. These results indicated that oral health status was poor in male patients, patients with low independence, patients with poor feeding status, patients with poor nutritional status, and patients with few teeth. The importance of oral health management for patients in convalescent hospitals was clearly observed.

**Table 6:** Multiple regression analysis (Objective variable: OHAT total score)

Explanatory Variables	B	SE	β	*p*-value	95% CI	VIF
Age	0.015	0.012	0.071	0.206	− 0.008 to 0.039	1.334
Sex*	−0.740	0.267	−0.143	0.006*	−1.265 to − 0.216	1.151
Stroke	0.050	0.314	0.009	0.875	−0.568 to 0.667	1.324
Bone fracture	0.118	0.310	0.021	0.704	−0.492 to 0.728	1.375
mRS	0.079	0.223	0.020	0.722	−0.360 to 0.519	1.376
CCI	0.066	0.085	0.039	0.436	−0.101 to 0.233	1.093
FOIS*	−0.347	0.095	−0.216	< 0.001*	− 0.534 to − 0.160	1.501
MNA-SF*	− 0.117	0.052	− 0.119	0.024*	−0.219 to − 0.015	1.185
Number of present teeth*	−0.048	0.015	−0.177	0.001*	−0.077 to − 0.020	1.232

*p < 0.05, Sex (0=man, 1=women) Stroke (0=not Stroke, 1=Stroke) Bone fractures (0=not Bone fractures, 1=Bone fractures)

## Discussion

This study investigated the association between ADL and oral health status at admission through a cross-sectional survey of all inpatients in the rehabilitation department of a convalescent hospital. The results showed that good swallowing function and oral health status were associated with high ADL at admission, in addition to the main disease, its severity, and nutritional status, suggesting the importance of oral health management during the convalescent period. In addition, poor oral health at admission was associated with low independence, poor feeding status, poor nutritional status, and fewer teeth, regardless of disease, suggesting the importance of oral health management during hospitalization in convalescent hospital for patients with these characteristics.

We found that most 322 patients over 65 years old were admitted to the convalescent hospital, and 235 of them had suffered strokes or fractures, which are often attributed to age, requiring long-term medical care [1]. Since the purpose of a convalescent hospital is to provide the ability to live independently at home or in a nursing home, it is important to prioritize ADL during hospitalization in a convalescent hospital. FIM scores shown in Table 1 indicate ADLs at the time of admission, showing relatively good cognitive function, although FIM motor and total scores were low. These trends were consistent with those of the previous studies [32, 33]. On the contrary, mRS, which indicates the severity of the disease, was higher than what was reported in previous studies [23], but this result may have been due to the higher number of stroke patients in this study. Regarding diet, while many patients followed a regular diet, the MNA-SF results revealed that several of them were malnourished. It is important to note that many people with DSS experience difficulty swallowing due to oral problems. The patients’ inability to consume an adequate diet and malnutrition can be attributed to oral health issues. It is worth noting that the OHAT score was low, indicating that, compared with previous studies, the oral health status of patients in this study was slightly better [8, 13] in acute and convalescent stages. Most patients had problems with oral functions such as dentures, saliva, natural teeth, and oral cleanliness. Based on previous studies, oral health management is often limited to oral hygiene management, especially in the acute phase, due to the short length of hospital stay. Therefore, in the convalescent period, oral health management focusing not only on oral hygiene but also on oral function should be considered essential [9].

In the univariate analysis of the correlation between the degree of independence and oral and general status, FIM motor, FIM cognitive, and FIM total score showed a significant moderate positive correlation with high values of DSS and FOIS and a significant weak negative correlation with high OHAT values. These results were consistent with those of previous studies [15]. Furthermore, considering that age, presence of stroke, presence of fracture, comorbidity, severity of the main disease, and nutritional status were also associated with FIM, we performed multiple regression analysis with these factors as explanatory variables. Multiple regression analysis with FIM total score, FIM motor, and FIM cognition as objective variables all showed the same trend results. The results showed that a high degree of independence was associated with non-stroke disease, less severe disease, good feeding status, good nutritional status, and good oral health, independently of each other. Oral health management is considered beneficial for those who have good motor function and can clean their own mouths, while those with poor motor function may not be able to clean their mouths adequately by themselves, resulting in poor oral health management. In addition, patients with impaired cognitive function may have difficulty understanding the importance of oral health management, making it difficult for them to adequately manage their oral health. In other words, the results suggest that improving both cognitive and motor functions may be related to maintaining and improving oral health management. Since many patients admitted to the hospital for recovery are older patients and it is difficult to improve their cognitive function, it may be important to improve their oral health status by improving their motor function. FIM is an assessment of ADL used as a discharge indicator in convalescent rehabilitation, and the fact that oral health status was associated with a high level of independence at admission to the convalescent period suggests the need for future medical-dental collaboration in the convalescent period. Because stroke patients are prone to sequelae, they tend to have a low level of independence, especially in the convalescent phase. However, it was clear that adequate improvement in oral health status was not achieved during acute hospitalization [34], emphasizing the importance of oral health management for improving ADL in stroke patients in their convalescent phase. Additionally, since good feeding status and nutritional status at the time of admission were associated with improved ADL, it was considered necessary to appropriately assess and manage oral functions necessary for oral intake, improve feeding status, and improve nutritional status through multidisciplinary collaboration with dieticians and other professionals. Furthermore, the FIM, which evaluates independence, has motor and cognitive aspects, both of which resulted in the same total score, suggesting that oral health status is related to both motor and cognitive aspects. A prior study has also found that cognitive decline is associated with poor oral cleaning [10], supporting the results of this study. The goal of convalescent rehabilitation is to improve independence with the goal of returning home, and the results of this study suggest the importance of oral health management during convalescence, as oral health status was associated with higher levels of independence upon admission. The results of this study support previous research in that the improvement of poor oral health through oral health management is associated with improvement in FIM [16].

In a multiple regression analysis using the OHAT total score as the objective variable, men, poor feeding status, poor nutritional status, and having few teeth were significantly associated with poor oral health status. The OHAT was developed for older adults prone to a comprehensive decline in oral function [31]. According to the results of this study, oral health management should be focused on hospitalized patients with poor feeding and nutritional status, regardless of their primary disease, and a previous study of stroke patients showed that poor oral health status was associated with poor feeding status [14, 15]. The OHAT is a simple assessment tool that can be used by healthcare professionals other than dentists. Therefore, for oral health management of patients hospitalized in convalescent hospitals, it was thought necessary to implement a medical-dental collaborative system in convalescent hospitals, in which nurses use the OHAT to evaluate the oral health status of patients upon admission and request oral health management from dentists according to their scores. In fact, in acute care hospitals, oral health management using the OHAT in collaboration with multiple professions has been shown to improve oral health and feeding status [9, 34]. Furthermore, since oral health management can be divided into oral hygiene management and oral function management, it was considered that not only improving oral hygiene by dental hygienists and daily oral care by nurses [10, 17] but also managing oral function by dentists, such as denture treatment, is essential.

This study has a few limitations. To eliminate any potential bias, gathering data from multiple centers would be beneficial rather than relying solely on the findings of this single-center study. Since it was necessary to narrow down the variables in this study due to the number of participants and the statistical analysis method, we are currently evaluating the oral status based on the number of teeth and OHAT, which has items for lip, tongue, gums and tissues, saliva, natural teeth, dentures, oral cleanliness, and dental pain, which are sufficient to evaluate the oral status. However, we would like to continue our research by increasing the number of oral indicators and investigating oral function, denture quality, tooth loss patterns, and the need for dental treatment. Additionally, because of the cross-sectional nature of this study, it is not known whether the subject’s level of independence improved during hospitalization or upon discharge. However, the results suggest that oral health care, which is difficult to provide in an acute setting, may improve the subject’s level of independence, nutritional status, and eating patterns during the recovery period. Longitudinal studies are needed to see changes in oral health management.

## Conclusions

A cross-sectional survey of all inpatients admitted to the rehabilitation department of a convalescent hospital revealed that high ADL at admission was associated with good swallowing function and good oral health status, in addition to non-stroke primary disease, low disease severity, and high nutritional status. These findings suggest the importance of oral health management during the convalescent period. In addition, poor oral health status at admission was associated with low independence, feeding status, poor nutritional status, and few teeth, regardless of disease, suggesting the importance of prioritizing oral health management for patients with these characteristics. We will continue to investigate and evaluate the patients at the time of discharge to elucidate longitudinally how oral improvements are related to their level of independence.

## Data Availability

The datasets generated and/or analyzed during the current study are not publicly available due to the need to protect the privacy of study participants, but are available from the corresponding author on reasonable request.

## Abbreviations

ADL: Activities of Daily Living
CCI: Charlson Comorbidity Index
FIM: Functional Independence Measure
mRS: modified Rankin Scale
FOIS: Functional Oral Intake Scale
DSS: Dysphagia Severity Scale
MNA-SF: Mini Nutritional Assessment-Short Form
OHAT: Oral Health Assessment Tool
